# Prediction models for acute kidney injury in patients with gastrointestinal cancers: a real-world study based on Bayesian networks

**DOI:** 10.1080/0886022X.2020.1810068

**Published:** 2020-08-25

**Authors:** Yang Li, Xiaohong Chen, Ziyan Shen, Yimei Wang, Jiachang Hu, Yunlu Zhang, Jiarui Xu, Xiaoqiang Ding

**Affiliations:** a Department of Nephrology, Zhongshan Hospital, Fudan University, Shanghai, China; b Shanghai Medical Center of Kidney, Shanghai, China; c Shanghai Key Laboratory of Kidney and Blood Purification, Shanghai, China; d Shanghai Institute of Kidney and Dialysis, Shanghai, China; e Hemodialysis Quality Control Center of Shanghai, Shanghai, China

**Keywords:** Gastrointestinal cancer, acute kidney injury, Bayesian network, Group LASSO, disease prediction, machine learning

## Abstract

**Background:**

This study attempts to establish a Bayesian networks (BNs) based model for inferring the risk of AKI in gastrointestinal cancer (GI) patients, and to compare its predictive capacity with other machine learning (ML) models.

**Methods:**

From 1 October 2014 to 30 September 2015, we recruited 6495 inpatients with GI cancers in a tertiary hospital in eastern China. Data on demographics, clinical and laboratory indicators were retrospectively extracted from the electronic medical record system. Predictors of AKI were selected in gLASSO regression, and further incorporated into BNs analysis.

**Results:**

The incidences of AKI in patients with esophagus, stomach, and intestine cancer were 20.5%, 13.9%, and 12.5%, respectively. Through gLASSO, 11 predictors were screened out, including diabetes, cancer category, anti-tumor treatment, ALT, serum creatinine, estimated glomerular filtration rate (eGFR), serum uric acid (SUA), hypoalbuminemia, anemia, abnormal sodium, and potassium. BNs model revealed that cancer category, treatment, eGFR, and hypoalbuminemia had direct connections with AKI. Diabetes and SUA were indirectly linked to AKI through eGFR, and anemia created connections with AKI through affecting album level. Compared with other ML models, BNs model maintained a higher AUC value in both the internal and external validation (AUC: 0.823/0.790).

**Conclusion:**

BNs model not only delineates the qualitative and quantitative relationship between AKI and its associated factors but shows the more robust generalizability in AKI prediction.

## Introduction

Gastrointestinal (GI) cancers, those of esophagus, stomach, colon, and rectum, are among the most common cancers worldwide. In China, 4.3 million new cancer cases were reported in 2015, of which over one-third belonged to GI cancers [1]. During anti-tumor therapy, kidney is one of the most vulnerable organs in early-stage, often leading to a poor prognosis and high in-hospital mortality. A Danish study reported that 1-year risk of acute kidney injury (AKI) among patients with rectum cancer was 25.1%, followed by colon cancer (22.5%), stomach cancer (19.9%), and esophagus cancer (19.8%) [2]. AKI in cancer patients was associated not only with advanced age and chronic comorbidities but with the tumor-specific factors, such as malignant infiltration, tumor lysis syndrome, nephrotoxic drugs, and interventional agents [3,4].

It is worth noting that 20 ∼ 30% AKI cases could be avoided if all risk factors were identified, quantified, and utilized for risk evaluation [5]. Previous studies created several logistic-score models to predict the AKI risk [6–9], while the predictive accuracy was usually questioned [10]. Till now, machine learning (ML) technique had been widely used to assist precision medicine [11,12]. Bayesian networks (BNs) is a framework for uncertainty reasoning, and it provides an intuitive depiction of the probabilistic structure for multivariate data. This character makes BNs suitable for large-scale data mining under the clinical conditions [13,14]. To this end, this study applied the Bayesian networks to establish a prediction model for inferring the risk of AKI in gastrointestinal cancer patients, and then compared its predictive capacity with other ML models.

## Methods

### Study design and patient recruitment

This study was designed as a real-world retrospective cohort study in a tertiary hospital in eastern China. Inpatients with GI cancers were recruited during 1 October 2014 and 30 September 2015. We excluded those who stayed less than 24 h, endured chronic kidney disease (CKD) stage 4–5, or underwent less than one test for serum creatinine (SCr). If the patient was hospitalized multiple times during the study period, we regarded each hospitalization as an independent case. Then the eligible participants were further randomized into two datasets by a ratio of 9:1. Ninety percent of them (*n* = 5845) was assigned as a derivation cohort for training the model, and the other 10% (*n* = 650) was assigned as a validation cohort for external validation (Supplementary Figure 1).

### Data collection

Data of this study were extracted from the electronic medical record (EMR) system. Limited by the indicator diversity and data missing, we only selected the most common clinical variables for analysis. It included age, gender, BMI, comorbidity, cancer category, and treatment. The baseline levels of biochemical indicators were set as the first test within 24 h after admission. Liver function was measured by alanine aminotransferase (ALT), aspartate aminotransferase (AST), and total bilirubin (TBiL). Renal function was measured by SCr, estimated glomerular filtration rate (eGFR), and serum uric acid (SUA). Other biochemical indicators were albumin, hemoglobin, serum sodium, and potassium. This study was approved by the ethics committee of Zhongshan Hospital, Fudan University (B2018-175). Each participant was assigned a unique code to replace their identity information.

### Definition and classification

AKI was diagnosed as a maximal increase in SCr by ≥0.3 mg/dL (26.5 μmol/L) within 48 h, or by ≥1.5 times baseline within the previous seven days [15]. Due to the inaccessibility of urine volume data, we dropped the urine volume changes to diagnose AKI. SCr measured on admission was considered as the baseline level. For patients who received multiple SCr tests during hospitalization, we used the highest value within seven days as the peak for AKI diagnosis. For patients who lacked baseline SCr but had regular follow-up visits in the past three months, we retrieved the mean value of outpatient SCr records as the baseline. The presence of hypertension and diabetes was determined by the diagnosis on admission and discharge records. Gastrointestinal cancer was categorized according to the international classification of diseases (ICD-10), which included esophagus cancer (C15), stomach cancer (C16), and intestine cancer (C17–21) [16]. Anti-tumor treatment in this study was grouped into surgery, chemotherapy, interventional therapy, and untreated/palliative care. The normal ranges of eGFR and SUA refer to ≥90 mL/min/1.73m^2^ and ≤359 μmol/L, respectively. Anemia is defined as a hemoglobin level <130 g/L in males and <120 g/L in females. Hypoalbuminemia refers to albumin <35 g/L. Hypo/hypernatremia was diagnosed if patients’ serum sodium level <137 mmol/L or >147 mmol/L. Similarly, hypo/hyperkalemia was defined when the potassium level was outside the normal ranges (3.5 ∼ 5.3 mmol/L).

### gLASSO regression

The least absolute shrinkage and selection operator (LASSO) regression can automatically select variables based on the tuning parameter value, and then shrink the estimates of irrelevant variables to zero [17]. In 2006, Yuan et al. [18] further proposed the gLASSO (group LASSO) regression as an extension. It overcomes the limitation of choosing the single dummy variable and can select the grouping variables as predefined. The gLASSO estimator is shown as ${\hat{\beta }}_{\lambda }=\arg_{\beta }m\mathrm{in}({\left||Y-X\beta |\right|}_{2}^{2}+\lambda \sum_{g=1}^{G}{\left||\beta_{l_{g}}|\right|}_{2}).$ β is the regression coefficient. The tuning parameter of λ controls the shrinkage degree. Tenfold cross-validation is used to choose the minimal λ value. The penalty of $l_{g}$ is the index set of gth grouping variables. It makes variable select at group levels and keep invariant under orthogonal transformations.

### Bayesian networks

BNs are composed of a set of random variables $X_{i}$ and a directed acyclic graph G = (V, A). Each node v∈V is associated with a variable $X_{i}.$ Directed arc a∈A represents the direct probabilistic dependencies. Others that are not linked by arcs are assumed as conditionally independent. The global distribution of X with parameters Θ can be factorized into: $P(X)=\prod_{i=1}^{n}P(X_{i}\pi (X_{i});\theta_{X_{i}}),$ where $\pi (X_{i})$ is the set of the parents of $X_{i}.$ Given its parents, each node $X_{i}$ is conditionally independent of its non-descendants. BNs establishment is performed as two steps: learning the structure and learning the local distribution. Structure learning includes constraint-based, score-based, and hybrid algorithms. Parameter learning is traced to maximum likelihood estimation and Bayesian estimation. Given the learned BNs model with structure G and parameters Θ, BNs reasoning can be converted into computing the posterior probabilities under a new piece of evidence E: P(XE,B)=P(XE,G,Θ).

### Statistical analysis

The homogeneity between derivation and validation cohorts were compared by using the Pearson test and Cochran–Mantel–Haenszel test. We applied the odds ratio (OR) and its 95% confidence interval (CI) to quantify the associations of possible risk factors with AKI. The gLASSO regression was used to select predictors of AKI among candidate variables, which were further presented to BNs analysis. Tabu-search algorithm was chosen to establish the BNs structure, and the maximum likelihood method was used to estimate the probability of each node according to the proposed BNs structure. We performed both internal and external validation to reduce model overfitting. Model evaluation indexes contained accuracy rate, recall rate, positive predictive value (PPV), negative predictive value (NPV), and the area under the receiver operating characteristic curve (AUC). Meanwhile, the BNs model was also compared horizontally with the models based on the decision tree, random forest (RF), support vector machine (SVM), naive Bayes, and logistic-score regression.

Statistical description and univariate analysis were performed in IBM SPSS (Version 22.0, IBM Corp., Armonk, NY, USA). The gLASSO and BNs analyses were run by using ‘grpreg’ and ‘bnlearn’ packages in R program (Version 3.6.0, R core team). The network structure was visualized in Netica (Version 5.18, Norsys Software Corp., Vancouver, BC, Canada). Other machine learning models were also created in R program with the packages of ‘tree’ (decision tree), ‘randomForest’ (RF), ‘e1071’ (SVM), ‘bnlearn’ (naive Bayes) and ‘glm’ (logistic-score model). The process of data cleaning and analysis was performed by the biostatistician (YL), and the full codes were attached in Supplement Text 1.

## Results

A total of 6495 participants were recruited in this study. The average age was 61.3 ± 11.8 years, and males accounted for 67.9%. Then we randomly divided them into the derivation cohort (*n* = 5845) and validation cohort (*n* = 650). Supplementary Table 1 suggested that the statistical distributions of most variables between the two cohorts were comparable (*p* > 0.05).

### AKI incidence and associated risk factors

**Figure 1.**
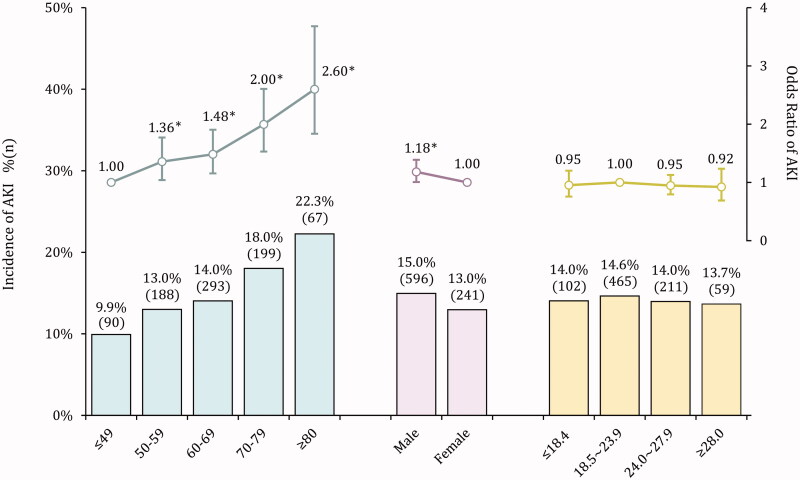
AKI incidence in varied demographics among patients with gastrointestinal cancers.

There were 837 AKI cases in the derivation cohort, with a pooled incidence of 14.3%. Of them, 783 patients located in AKI stage 1 (93.5%), and 54 patients developed to stage 2–3. The proportion of renal replacement therapy was 2.7% (*n* = 23). The AKI incidence increased along with advancing age, from 9.9% in the youngest to 22.3% in patients over 80 years old (Figure 1). Patients with esophagus cancer shared the highest AKI incidence (20.5%), followed by stomach cancer (13.9%) and intestine cancer (12.5%). After adjusting for the demographic factors, we found that patients admitted with hypertension and diabetes (aOR: 1.15/1.66) or in an emergency (aOR: 1.38) were at increased risk of AKI (Table 1). Compared with untreated/palliative care, patients who underwent surgery were more vulnerable to AKI (aOR: 8.87). Other factors associated with AKI contained liver dysfunction (aOR: 1.34 ∼ 2.07), poor eGFR (aOR: 1.98 ∼ 8.82), high SUA level (aOR: 1.35 ∼ 3.29), hypoalbuminemia (aOR: 2.08), anemia (aOR: 2.75) and electrolyte abnormities (aOR: 2.23 ∼ 8.99).

**Table 1.** Risk factors of AKI in patients with gastrointestinal cancers in the derivation cohort.

Variable	Total	AKI (%)	χ^2^	*p* -value	cOR (95%CI)	aOR (95%CI)^a^
Comorbidities						
Hypertension	683	118 (17.3)	5.511^†^	0.019	1.29 (1.04∼1.60)	1.15 (0.92∼1.44)
Diabetes	348	78 (22.4)	19.757^†^	<0.001	1.80 (1.39∼2.35)	1.66 (1.27∼2.17)
Cancer category						
Esophagus	908	186 (20.5)	35.104^†^	<0.001	1.80 (1.47∼2.20)	1.83 (1.49∼2.25)
Stomach	2453	340 (13.9)			1.12 (0.95∼1.33)	1.14 (0.97∼1.35)
Intestine	2484	311 (12.5)			1.00	1.00
Cancer stage						
Loco-regional	5338	777 (14.6)	2.796^†^	0.095	0.79 (0.60∼1.04)	0.78 (0.59∼1.03)
Metastases	507	60 (11.8)			1.00	1.00
In-hospital condition						
Emergent	554	107 (19.3)	12.441^†^	<0.001	1.50 (1.19∼1.87)	1.38 (1.10∼1.74)
Normal	5291	730 (13.8)			1.00	1.00
Treatment						
Surgery	2859	640 (22.4)	301.444^†^	<0.001	7.34 (3.99∼13.50)	8.87 (4.79∼16.44)
Chemotherapy	2179	162 (7.4)			2.04 (1.10∼3.81)	2.40 (1.28∼4.50)
Interventional	516	24 (4.7)			1.24 (0.60∼2.57)	1.49 (0.72∼3.11)
Untreated/palliative	291	11 (3.8)			1.00	1.00
Liver function						
ALT (≥80 U/L)	311	76 (24.4)	27.404^†^	<0.001	2.03 (1.55∼2.66)	2.07 (1.58∼2.72)
AST (≥70 U/L)	272	52 (19.1)	5.352^†^	0.021	1.44 (1.06∼1.97)	1.42 (1.04∼1.95)
TBiL (≥20.4 μmol/L)	508	93 (18.3)	7.209^†^	0.007	1.38 (1.09∼1.76)	1.34 (1.05∼1.70)
Renal function						
SCr (≥115 μmol/L)	209	118 (56.5)	313.701^†^	<0.001	8.87 (6.67∼11.79)	7.93 (5.93∼10.60)
eGFR (≥90 mL/min/1.73m^2^)	3263	302 (9.3)	282.507^‡^	<0.001	1.00	1.00
eGFR (60∼89 mL/min/1.73m^2^)	2282	390 (17.1)			2.02 (1.72∼2.37)	1.98 (1.67∼2.33)
eGFR (≤59 mL/min/1.73m^2^)	300	145 (48.3)			9.17 (7.10∼11.84)	8.82 (6.73∼11.56)
SUA (≤359 μmol/L)	4670	589 (12.6)	91.391^‡^	<0.001	1.00	1.00
SUA (360∼420 μmol/L)	709	117 (16.5)			1.37 (1.10∼1.70)	1.35 (1.08∼1.69)
SUA (421∼480 μmol/L)	300	76 (25.3)			2.35 (1.79∼3.09)	2.25 (1.70∼2.98)
SUA (≥481 μmol/L)	166	55 (33.1)			3.43 (2.46∼4.80)	3.29 (2.34∼4.62)
Biochemical test						
Hypoalbuminemia	1960	416 (21.2)	114.578^†^	<0.001	2.22 (1.91∼2.57)	2.08 (1.79∼2.42)
Anemia	4205	725 (17.2)	104.251^†^	<0.001	2.84 (2.31∼3.50)	2.75 (2.23∼3.40)
Hyponatremia	1290	352 (27.3)	293.274^†^	<0.001	3.39 (2.90∼3.96)	3.28 (2.80∼3.84)
Hypernatremia	113	42 (37.2)			5.34 (3.60∼7.92)	5.08 (3.41∼7.56)
Hypokalemia	796	187 (23.5)	206.422^†^	<0.001	2.24 (1.86∼2.69)	2.23 (1.85∼2.69)
Hyperkalemia	90	51 (56.7)			9.52 (6.22∼14.57)	8.99 (5.86∼13.81)

^a^aOR was adjusted for age, gender, and body mass index. ^†^refers to Pearson test (for binary and unordered categorical variables). ^‡^refers to Cochran–Mantel–Haenszel test (for ordered categorical variables).

AKI: Acute kidney injury; cOR: crude Odds ratio; aOR: adjusted Odds ratio; ALT: Alanine aminotransferase; AST: Aspartate aminotransferase; TBiL: Total Bilirubin; SCr: Serum creatinine; eGFR: estimated Glomerular filtration rate; SUA: Serum uric acid.

### Variable selection in gLASSO regression

When the tuning parameter log(λ) was set at −4.837, 11 predictors with non-zero coefficients were selected through gLASSO regression (Supplementary Figure 2). It included diabetes, cancer category, treatment, ALT, SCr, eGFR, SUA, hypoalbuminemia, anemia, abnormal sodium, and potassium levels. Then we incorporated these variables into logistic analysis to quantify their associations with AKI (Table 2). In the multivariate model, the major contributors of AKI were esophagus cancer, surgery, poor eGFR and electrolyte disorders, with an OR ranged from 1.78 to 8.52.

**Table 2.** Predictive variables of AKI selected by gLASSO regression.

Variate	OR (95% CI)	*p* -value	
Diabetes	1.64 (1.21∼2.24)	0.002	
Cancer Category (Esophagus)	2.83 (2.24∼3.58)	<0.001	
Cancer Category (Stomach)	1.28 (1.07∼1.54)	0.008	
Treatment (Surgery)	8.52 (4.39∼16.55)	<0.001	
Treatment (Chemotherapy)	2.23 (1.14∼4.38)	0.019	
Treatment (Interventional)	2.04 (0.93∼4.47)	0.074	
ALT (≥80 U/L)	1.79 (1.32∼2.44)	<0.001	
SCr (≥115 umol/L)	3.57 (2.01∼6.35)	<0.001	
eGFR (60∼89 mL/min/1.73m^2^)	1.81 (1.51∼2.16)	<0.001	
eGFR (≤59 mL/min/1.73m^2^)	4.04 (2.42∼6.76)	<0.001	
SUA (360∼420 μmol/L)	1.09 (0.85∼1.40)	0.501	
SUA (421∼480 μmol/L)	1.79 (1.28∼2.51)	0.001	
SUA (≥481 μmol/L)	1.79 (1.16∼2.77)	0.008	
Hypoalbuminemia	1.26 (1.06∼1.50)	0.008	
Anemia	1.61 (1.28∼2.02)	<0.001	
Hyponatremia	2.17 (1.82∼2.59)	<0.001	
Hypernatremia	3.34 (2.14∼5.22)	<0.001	
Hypokalemia	1.78 (1.44∼2.18)	<0.001	
Hyperkalemia	2.52 (1.53∼4.14)	<0.001	

gLASSO: group LASSO; OR: Odds ratio; ALT: Alanine aminotransferase; SCr: Serum creatinine; eGFR: estimated Glomerular filtration rate; SUA: Serum uric acid.

### BNs establishment and probabilistic reasoning

**Figure 2.**
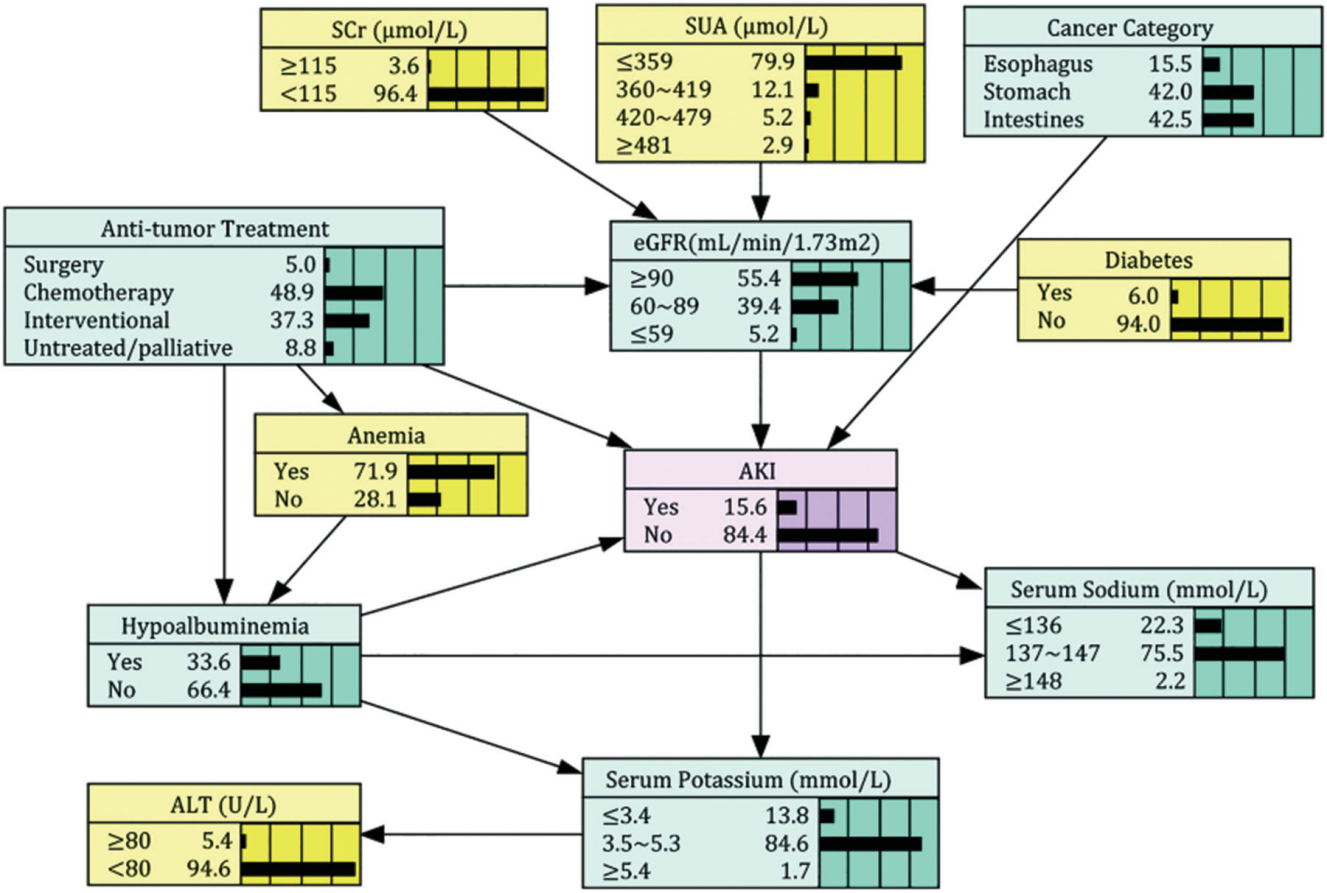
Bayesian Network model of AKI risk factors in patients with gastrointestinal cancers. AKI: Acute kidney injury; ALT: Alanine aminotransferase; SCr: Serum creatinine; eGFR: estimated Glomerular filtration rate; SUA: Serum uric acid.

Figure 2 was the AKI probabilistic model established by Bayesian networks. It consisted of 12 nodes and 17 arcs. Each node represented one variable, and the arc between connected nodes indicated the probabilistic dependencies. The marginal probability of AKI was estimated at 15.6%. Cancer category, treatment, hypoalbuminemia, and eGFR had direct connections with AKI, while serum potassium and sodium acted as the child nodes of AKI. Diabetes and SUA were indirectly linked to AKI through eGFR, and anemia created connections with AKI through affecting the album level. Correlations between predictors were also exhibited in the network: treatment was associated with hypoalbuminemia, anemia, and eGFR; hypoalbuminemia directly linked with serum potassium and sodium. Given the limited evidence variables, BNs model can compute the maximum *a posteriori* of AKI through probabilistic reasoning. For instance, when one patient was detected with esophagus cancer, hyperuricemia, anemia, and hyponatremia at admission, the AKI risk was estimated at 51.9%. In contrast, for patients with similar diagnosis but without biochemical abnormities, the incidence of AKI was only 13.9% (Supplementary Figure 3).

### Predictive ability comparison between BNs model and other ML models

**Figure 3.**
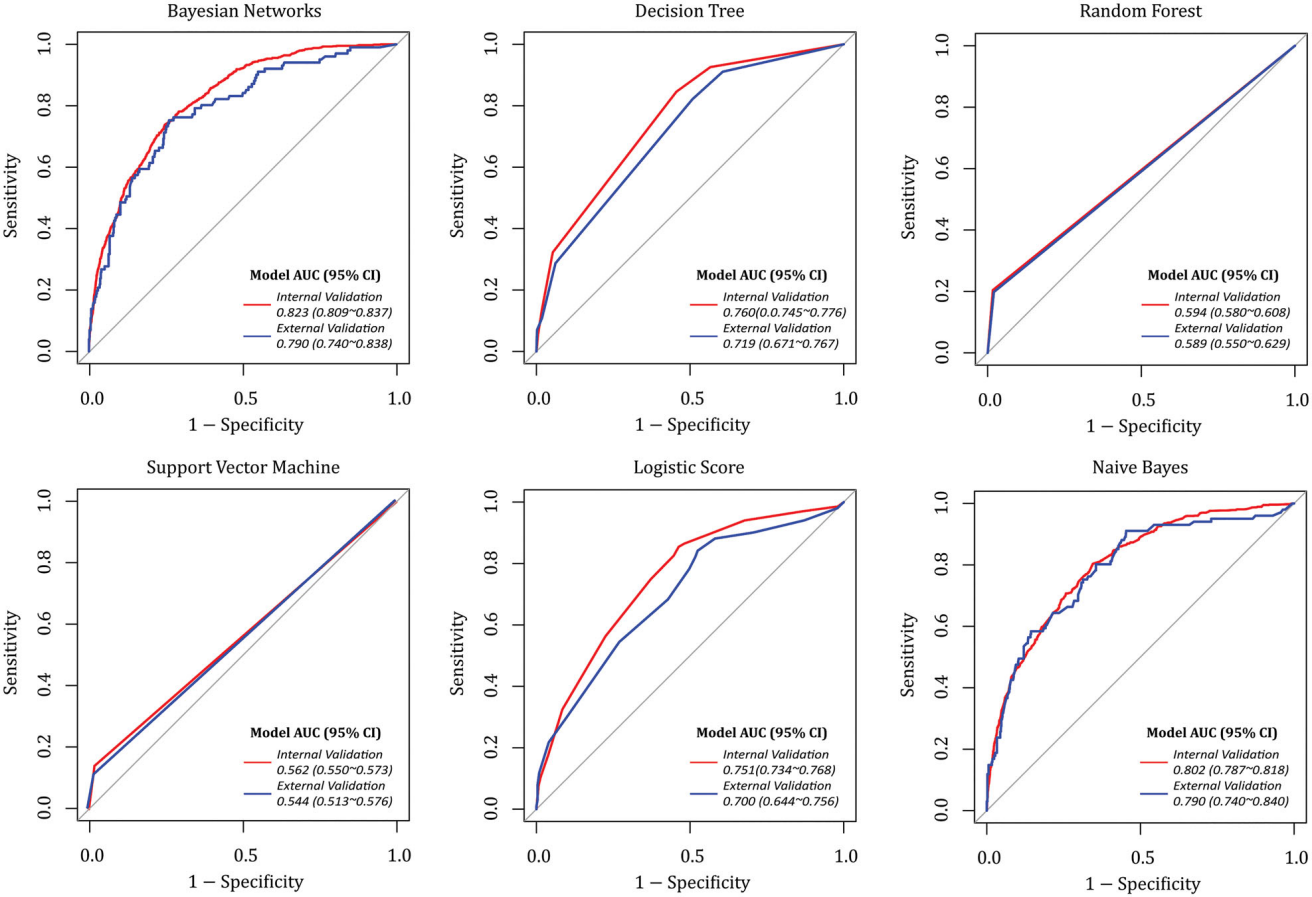
Receiver operating characteristic curves of AKI prediction models based on different ML algorithms.

ROC curves of prediction models based on different ML algorithms were drawn in Figure 3. In internal validation, the AUC values ranked from high to low were the BNs (0.823), naive Bayes (0.802), decision tree (0.760), logistic score (0.751), RF (0.594) and SVM (0.562). DeLong’s test verified that AUC value of BNs model is significantly higher than that of other ML models (*p* < 0.05 in naive Bayes; *p* < 0.001 in other MLs). Although the accuracy rates in ML models were over 85%, the models’ precision differed in a large margin (Table 3). Of them, the BNs model could possess a comparably better agreement between the actual observations and the predictions. According to internal validation, about 60.7% and 88.8% predicted cases belonged to true positives/negatives in BNs model.

**Table 3.** Internal and external validation of AKI prediction models based on different ML algorithms.

Models	Internal validation				External validation			
	Accuracy	Recall	PPV	NPV	Accuracy	Recall	PPV	NPV
Bayesian Network	87.0%	26.5%	60.7%	88.8%	85.1%	24.8%	54.3%	87.4%
Decision tree	86.1%	13.5%	56.2%	87.2%	84.6%	10.9%	52.4%	85.7%
Random forest	87.3%	22.1%	67.3%	88.3%	85.5%	19.8%	60.6%	86.9%
Support vector machine	86.2%	14.1%	56.7%	87.2%	84.5%	10.9%	50.0%	85.7%
Logistic-score	85.7%	7.4%	65.3%	86.1%	85.4%	7.9%	80.0%	85.5%
Naive Bayes	86.2%	30.5%	53.3%	89.2%	84.9%	28.7%	52.7%	87.9%

Accuracy rate is the sum of correctly classified cases test divided by the data set size (TP + TN)/(TP + TN + FP + FN). Recall rate is the positively classified cases divided by the positive cases TP/(TP + FN). Positive predictive value (PPV) is the proportion of positive cases that are true positives TP/(TP + FP). Negative predictive value (NPV) is the proportion of negative cases that are true negatives TN/(TN + FN).

## Discussion

Gastrointestinal cancer remains a global health problem with an estimated 3.4 million new diagnosed cases in 2018 worldwide [19]. During the prolonged anti-tumor treatment, patients were exposed to higher risks of complications, including AKI. It was reported that about 50.0% of AKI cases and 18.8% of AKI deaths occurred in patients with GI cancer [20]. Differed from previous studies (2.4%∼35.3%) [21–24], the AKI incidence of esophagus, stomach, and intestine cancer in this study was 20.5%, 13.9%, and 12.5%, respectively. It may be partly explained by the heterogeneity of source population, the different definitions for AKI as well as oncologists’ neglect of AKI diagnosis.

In the pathogenesis of AKI, factors do not exist separately, but are closely related. The association between one factor and AKI may be affected by the presence of other covariates. LASSO, based on the penalization and regularization techniques, was adept in processing such higher-dimensional data. In this study, we applied gLASSO regression to select the 11 most significant predictors of AKI before modeling. It simplifies the complexity of network and avoids overfitting and misclassification. Other studies also proved LASSO/gLASSO as an effective tool for variable selection in ML modeling [25–27].

It is the first time to apply BNs to graphically present the probabilistic dependencies between AKI and its associated factors in GI cancer patients. As shown in Figure 2, direct linkages to AKI were observed in anti-tumor treatment, cancer category, eGFR, and hypoalbuminemia. Either partial or total surgical excision of the lesion triggers a series of ischemia-reperfusion injuries, activation of inflammatory mediators and complement, production of free radicals, etc. These changes can further reduce renal perfusion and induce cell injury or death. Moreover, many anti-tumor drugs had potential nephrotoxicity [28]. It was reported that 80.1% of cancer patients had received such drugs [29]. Hypoalbuminemia is also commonly encountered in GI cancer patients, which could be caused by nutritional deficiencies (malnutrition, malabsorption) and volume depletion (diarrhea, vomiting, and drainage of ascites) [30]. Volume depletion disturbs the cellular environmental homeostasis, leading to electrolyte disorders [31]. Still, there was a small proportion of patients admitted with hypernatremia and hyperkalemia. It may be caused by the improper use of chemotherapy agents. Findings from our study revealed that both low and high electrolyte levels could increase the AKI risk. Gao et al. [32] also found that, of AKI patients, 27.0% and 5.7% were admitted with hypo-/hypernatremia; 16.6% and 24.4% were with hypo-/hyperkalemia.

Given the limited evidence variables, BNs model can compute the risk of AKI based on uncertain inference. While in the traditional logistic model, it can be done only if we knew the state of all variables. From a health economic point of view, BNs seems to provide a more practicable strategy to identify high-risk patients. Additionally, BNs model also maintained a greater AUC value in both the internal and external validation (AUC: 0.823/0.790) than other ML models. Rohit et al. [33] compared three ML models for AKI detection and found that naive Bayes performed better (AUC: 0.699). Lee et al. [34] created a gradient boosting model for predicting cardiac surgery-associated AKI, and the AUC value reached 0.780. Briefly, decision tree is an instance-based learning method, which can induce tree model from training samples. RF is based on ensemble leaning algorithm. It constructs many decision trees to make a vote on classification results. SVM is based on supervised learning algorithm. It can minimize the empirical error and maximize the geometric margin at the same time. However, if solely using clinical risk factors but without specific AKI markers, model’s predictive precision will be challenged. For this reason, we found that in BNs model, the recall rate and PPV were limited to 26.5% and 60.7%. Yet even so, integrating BNs model into the healthcare can still assist in identifying the high-risk patients at an early stage (before SCr rising), and increase their chances of AKI treatment [35].

This study has several limitations to declare. Firstly, our study was conducted in a single center. The extrapolation of results may be influenced by selection bias. Secondly, we only selected the clinical variables with the least data missing. Other factors, such as hemorrhage, infection or nephrotoxic drugs, were not enrolled. It will blur the relationships with unknown variables and AKI to some extent. Thirdly, data were collected retrospectively. Arcs in the BNs model only represented the probabilistic dependency relationships between variables, and their causal relationships need to be further verified. In further studies, novel AKI biomarkers, such as KIM-1, NGAL, and TIMP-2*IGFBP7, were considered to retrofit into model and improve its predictive precision.

In conclusion, AKI remains a higher incidence among GI patients. BNs model not only delineates the qualitative and quantitative relationship between AKI and its associated factors, but shows the more robust generalizability in AKI prediction. It can help physicians to identify patients at high risks for AKI and take preventive strategies to improve prognosis.

## Supplementary Material

Supplemental Material
