# Supplementary material for: Prediction models for acute kidney injury in patients with gastrointestinal cancers: a real-world study based on Bayesian networks
Source: Ren Fail. 2020 Aug 25;42(1):869–76. doi: 10.1080/0886022X.2020.1810068 (PMC7472473; doi:10.1080/0886022X.2020.1810068)
Supplement: Supplemental Material [file IRNF_A_1810068_SM4562.pdf]

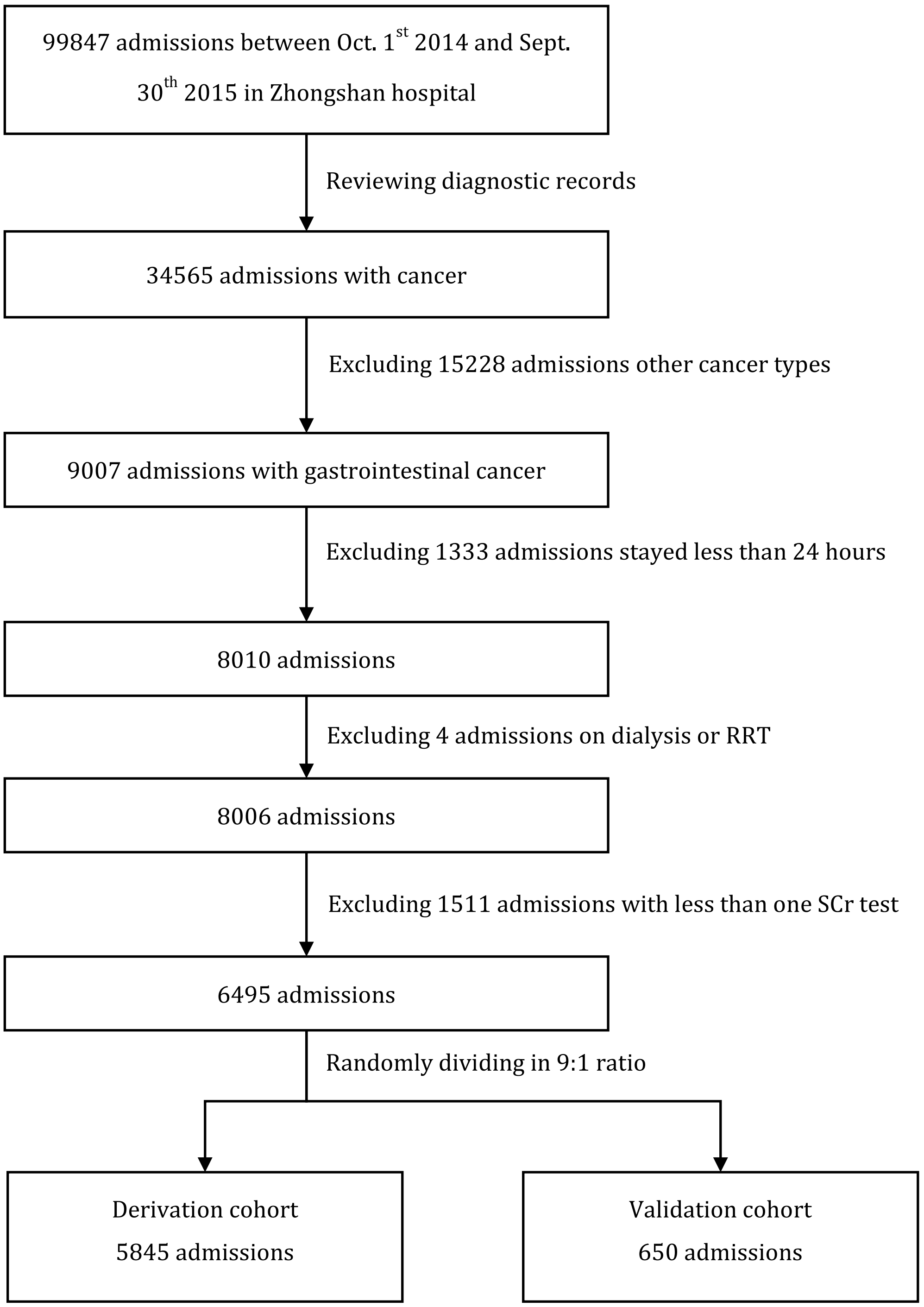

Supplement Figure 1. Flow chart of the study population selection

Supplement Table 1. Demographic and clinical features in derivation and validation cohorts

Variate	Derivation cohort (n=5845)	Validation cohort (n=650)	χ^2	p-value
**AKI**				
Yes	837(14.3)	103(15.5)	0.703	0.402
No	5008(85.7)	549(84.5)		
**Age**				
<49 yr	907(15.5)	91(14.0)	1.678	0.795
50~59 yr	1446(24.7)	162(24.9)		
60~69 yr	2087(35.7)	230(35.4)		
70~79 yr	1104(18.9)	133(20.5)		
≥80 yr	301(5.1)	34(5.2)		
**Gender**				
Male	3985(68.2)	423(65.1)	2.579	0.108
Female	1860(31.8)	227(34.9)		
**BMI**				
<18.4	726(12.4)	73(11.2)	9.282	0.026
18.5~23.9	3176(54.3)	356(54.8)		
24.0~27.9	1511(25.9)	152(23.4)		
≥28.0	432(7.4)	69(10.6)		
**Comorbidities**				
Hypertension	683(11.7)	74(11.4)	0.051	0.821
Diabetes	348(6.0)	36(5.5)	0.181	0.670
**Cancer Stage**				
Loco-regional	5338(91.3)	598(92.0)	0.338	0.561
Metastases	507(8.7)	52(8.0)		
**Cancer Position**				
Esophagus	908(15.5)	92(14.2)	3.532	0.171
Stomach	2453(42.0)	257(39.5)		
Intestine	2484(42.5)	301(46.3)		
**In-hospital Condition**				
Emergent	554(9.5)	54(8.3)	0.945	0.331
Normal	5291(90.5)	596(91.7)		
**Treatment**				
Surgery	2859(48.9)	349(53.7)	6.693	0.082
Chemotherapy	2179(37.3)	217(33.4)		
Interventional	516(8.8)	59(9.1)		
Untreated/palliative	291(5.0)	25(3.8)		
**Liver Function**				
ALT (≥80 U/L)	311(5.3)	33(5.1)	0.069	0.792
AST (≥70 U/L)	272(4.7)	38(5.8)	1.830	0.176
TBiL(≥20.4 μmol/L)	508(8.7)	58(8.9)	0.040	0.842
**Renal Function**				
SCr(≥115 μmol/L)	209(3.6)	22(3.4)	0.062	0.803
eGFR(≥90 mL/min/1.73m²)	3263(55.8)	355(54.6)	0.395	0.821
eGFR(60~89 mL/min/1.73m²)	2282(39.0)	262(40.3)		
eGFR(≤59 mL/min/1.73m²)	300(5.1)	33(5.1)		
SUA(≤359 μmol/L)	4670(79.9)	516(79.4)	6.240	0.100
SUA(360~420 μmol/L)	709(12.1)	82(12.6)		
SUA(421~480 μmol/L)	300(5.1)	42(6.5)		
SUA(≥481 μmol/L)	166(2.8)	10(1.5)		
**Biochemical Test**				
Hypoalbuminemia	1960(33.5)	245(37.7)	4.513	0.034
Anemia	4205(71.9)	461(70.9)	0.300	0.584
Hyponatremia	1290(22.1)	153(23.5)	1.246	0.536
Hypernatremia	113(1.9)	15(2.3)		
Hypokalemia	796(13.6)	83(12.8)	0.433	0.805
Hyperkalemia	90(1.5)	11(1.7)		

AKI: Acute kidney injury; BMI: Body mass index; ALT: Alanine aminotransferase; AST: Aspartate aminotransferase; TBiL: Total Bilirubin; SCr: Serum creatinine; eGFR: estimated Glomerular filtration rate; SUA: Serum uric acid.

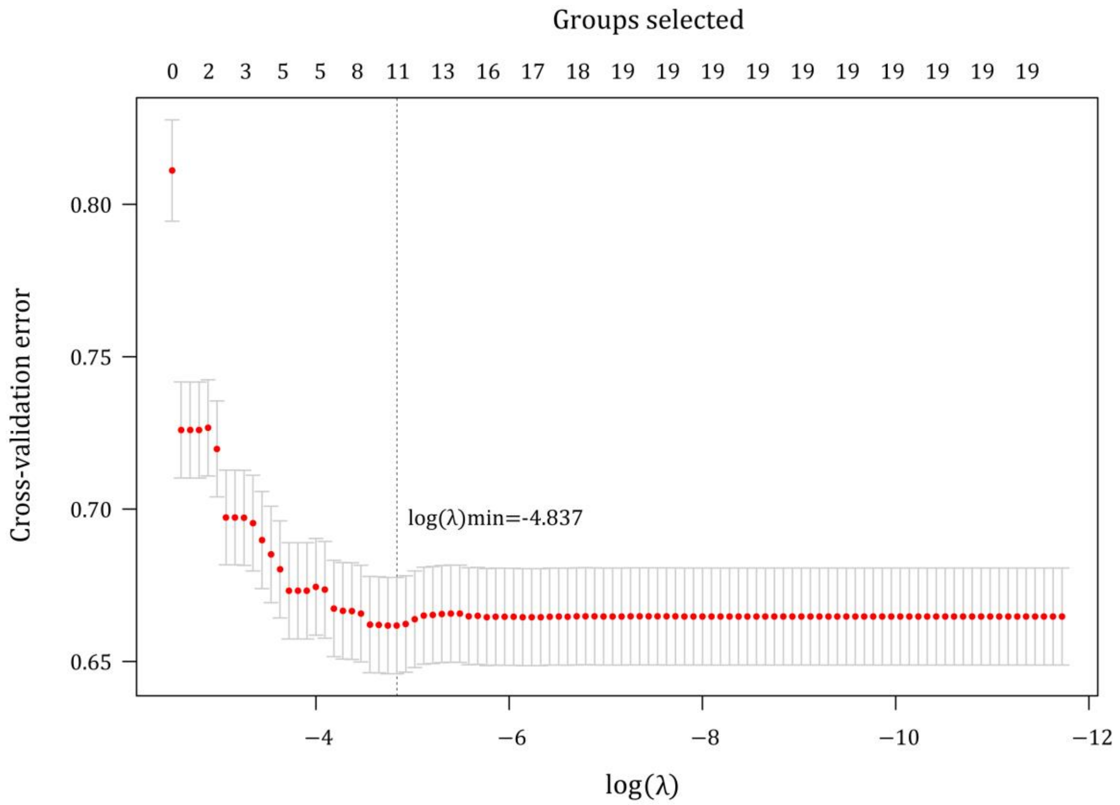

Supplement Figure 2. AKI candidate variable selection by using LASSO regression

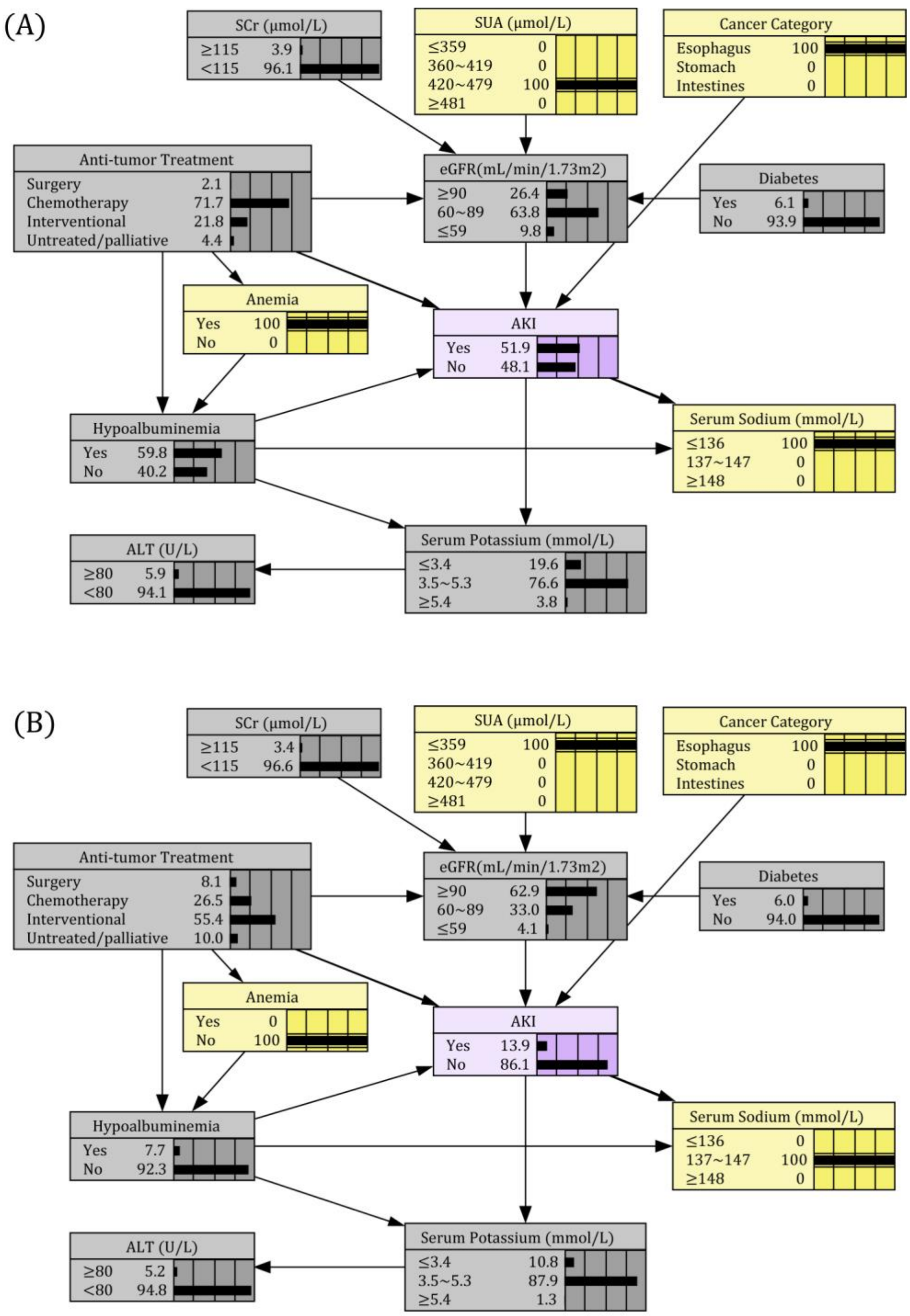

Supplement Figure 3. Bayesian network inference in the given evidence variables (3A: AKI incidence was estimated in given evidence variables of cancer category, hemoglobin, serum uric acid and serum sodium. 3B: AKI incidence was estimated in patients with similar diagnosis but without biochemical abnormities.)

AKI: Acute kidney injury; ALT: Alanine aminotransferase; SCr: Serum creatinine; eGFR: estimated Glomerular filtration rate; SUA: Serum uric acid.

Supplement Text 1. R codes to perform BNs and machine learning techniques.

```
#------- Data loading -------#

setwd("C:\\Users\\Desktop")
aki <- read.csv(file="aki.csv")
n <- dim(aki)[1]
p <- dim(aki)[2]
n1 <- table(aki$AKI)[1]
n2 <- table(aki$AKI)[2]

#---- Making derivation and validation data sets ----#

set.seed(2020)
head(aki)
sample1 <- sample(which(aki$AKI==0), round(n1/10), replace=F)
sample2 <- sample(which(aki$AKI==1), round(n2/10), replace=F)
dc_sample <- c(sample1,sample2)
aki_dc <- aki[dc_sample,]
aki_vc <- aki[-dc_sample,]

#------Transfer the data format from "numeric" to "factor"-----#

for (i in 1:n) {
   aki_dc[,i] <- as.factor(aki_dc[,i])
}
summary(aki_dc)

for (i in 1:n) {
   aki_vc[,i] <- as.factor(aki_vc[,i])
}
summary(aki_vc)

#------AKI candidate variable selection by using LASSO regression-----#

install.packages("grpreg")
library(grpreg)

aki_glasso <- read.csv(file="aki_glasso.csv")
group=as.factor(c("AGE", "AGE", "AGE", "AGE","GENDER","BMI","BMI","BMI","HBP",
"DM","SITE","SITE","ZY","INHOS","TREAT", "TREAT","TREAT","AST", "ALT","TBIL", "SCR",
"GFR","GFR", "UA", "UA", "UA", "ALB", "HGB","WBC","N","N","K","K"))
x <- as.matrix(aki_glasso[,1:33])
y <- aki_glasso$AKI
```

```
set.seed(2020)

fit <- cv.grpreg(x, y, group, penalty="grLasso",
family="binomial",nfolds=10,returnY=FALSE, trace=FALSE)
plot(fit,bty="o")
exp(coef(fit))
log(fit$lambda.min)

aki_dc_lasso <- read.csv(file="aki_dc_lasso.csv")
for (i in 1:12) {
  aki_dc_lasso[,i] <- as.factor(aki_dc_lasso[,i])
}
str(aki_dc_lasso)

logit_form = as.formula(paste("aki_dc_lasso$AKI~ ",paste(names(aki_dc_lasso[,-
1]),collapse='+')))
logit_grlasso <- glm(logit_form,data=aki_dc_lasso,family=binomial(link = "logit"))
exp(logit_grlasso$coefficients); exp(confint(logit_grlasso))

#------Bayesian networks modeling-----#

install.packages(c("bnlearn","Rgraphviz","gRain","ggplot2","ROCR"))

library("bnlearn")
library("Rgraphviz")
library("ggplot2")
library("ROCR")

# structure learning
aki_dc_bn <- read.csv(file="aki_dc_lasso_bn.csv")

bn_structure <- tabu(aki_dc_bn, score = "bic", tabu = 10, max.tabu = 5, max.iter = Inf, maxp
= Inf, optimized = TRUE)
graphviz.plot(bn_structure)
bn_structure

# paramater learning
bn_model <- bn.fit(bn_structure,aki_dc_bn, method = "mle")

# BNs model inference
cpquery(bn_model, event = (AKI == "A1"),evidence = ( SITE== "A1")&(UA == "A2")&(HGB
== "A1")&(Na == "A0"), method = "ls", n = 10^6)
cpquery(bn_model, event = (AKI == "A1"),evidence = ( SITE== "A1")&(UA == "A0")&(HGB
== "A0")&(Na == "A1"), method = "ls", n = 10^6)
```

```
#compute error rate
evidence_dc <- data.frame(aki_dc_bn)
pred_dc_err <- predict(bn_model, "AKI", evidence_dc, method = "bayes-lw",prob = TRUE)

table(aki_dc_bn$AKI,pred_dc_err)
error_rate_dc <- mean(aki_dc_bn[,1]!= pred_dc_err)
print(error_rate_dc)

# estimate ROC and AUC
prob_dc <- t(attr(pred_dc_err, "prob"))[,2]
library(pROC)
roc_dc_bn <- roc(aki_dc_bn$AKI, prob_dc)
print(roc_dc_bn)
ci(roc_dc_bn)
plot_roc_dc_bn <- plot.roc(roc_dc_bn, col="red",print.auc=FALSE, max.auc.polygon=FALSE,
                           print.thres=FALSE,auc.polygon=FALSE,legacy.axes=TRUE)

#error rate and AUC in validation cohort dataset
aki_vc_bn <- read.csv(file="aki_vc_lasso_bn.csv")
evidence_vc <- data.frame(aki_vc_bn)
pred_vc_err <- predict(bn_model, "AKI", evidence_vc, method = "bayes-lw",prob = TRUE)

table(aki_vc_bn$AKI,pred_vc_err)
error_rate_vc <- mean(aki_vc_bn[,1]!= pred_vc_err)
print(error_rate_vc)

prob_vc <- t(attr(pred_vc_err, "prob"))[,2]
roc_vc_bn <- roc(aki_vc_bn$AKI, prob_vc)
print(roc_vc_bn)
ci(roc_vc_bn)
plot_roc_vc_bn <- plot.roc(roc_vc_bn, col="blue",print.auc=FALSE, max.auc.polygon=FALSE,
                           print.thres=FALSE,auc.polygon=FALSE,legacy.axes=TRUE,add
= TRUE)


#------Decision Tree Classification -----#

install.packages("tree")
library(tree)

aki_dc_ml <- read.csv(file="aki_dc_lasso_ml.csv")
aki_vc_ml <- read.csv(file="aki_vc_lasso_ml.csv")
```

```
for (i in 1:12) {
  aki_dc_ml[,i] <- as.factor(aki_dc_ml[,i])
}
str(aki_dc_ml)

for (i in 1:12) {
  aki_vc_ml[,i] <- as.factor(aki_vc_ml[,i])
}
str(aki_vc_ml)

#creating decision tree model
aki_tree = tree(factor(AKI) ~., aki_dc_ml)
summary(aki_tree)
aki_tree

cv_aki_tree <- cv.tree(aki_tree)0
plot(cv_aki_tree$size, cv_aki_tree$dev, type='b')

aki_tree_prun <- prune.tree(aki_tree,best=6)
plot(aki_tree_prun)
text(aki_tree_prun,pretty=0)

#error rate
error_rate_dc_tree<- predict(aki_tree_prun, data=aki_dc_ml[,-1],type="class")
mean(aki_dc_ml$AKI != error_rate_dc_tree)
table(aki_dc_ml$AKI, error_rate_dc_tree)

error_rate_vc_tree<- predict(aki_tree_prun, newdata=aki_vc_ml[,-1],type="class")
mean(aki_vc_ml$AKI != error_rate_vc_tree)
table(aki_vc_ml$AKI, error_rate_vc_tree)

#ROC and AUC
error_rate_dc_tree2<- predict(aki_tree_prun, data=aki_dc_ml[,-1])
error_rate_vc_tree2<- predict(aki_tree_prun, newdata=aki_vc_ml[,-1])

roc_dc_tree <- roc(aki_dc_ml$AKI, error_rate_dc_tree2[,2])
print(roc_dc_tree)
ci(roc_dc_tree)
plot_dc_tree <- plot.roc(roc_dc_tree, col="red",print.auc=FALSE, max.auc.polygon=FALSE,
                         print.thres=FALSE,auc.polygon=FALSE,legacy.axes=TRUE)

roc_vc_tree <- roc(aki_vc_ml$AKI, error_rate_vc_tree2[,2])
print(roc_vc_tree)
ci(roc_vc_tree)
```

```
plot_vc_tree <- plot.roc(roc_vc_tree, col="blue",print.auc=FALSE, max.auc.polygon=FALSE,
                         print.thres=FALSE,auc.polygon=FALSE,legacy.axes=TRUE,add
= TRUE)

#------Random Forest Regression -----#

install.packages("randomForest")
library(randomForest)

#creating random forest model
aki_rf <- randomForest(AKI~.,data=aki_dc_ml,importance=TRUE)
aki_rf
importance(aki_rf)
varImpPlot(aki_rf)

#error rate
error_rate_dc_rf<- predict(aki_rf, data=aki_dc_ml[,-1],type="class")
mean(aki_dc_ml$AKI != error_rate_dc_rf)
table(aki_dc_ml$AKI, error_rate_dc_rf)

error_rate_vc_rf<- predict(aki_rf, newdata=aki_vc_ml[,-1],type="class")
mean(aki_vc_ml$AKI != error_rate_vc_rf)
table(aki_vc_ml$AKI, error_rate_vc_rf)

#ROC and AUC
roc_dc_rf <- roc(aki_dc_ml$AKI, as.numeric(error_rate_dc_rf))
print(roc_dc_rf)
ci(roc_dc_rf)
plot_dc_rf <- plot.roc(roc_dc_rf, col="red",print.auc=FALSE, max.auc.polygon=FALSE,
                       print.thres=FALSE,auc.polygon=FALSE,legacy.axes=TRUE)

roc_vc_rf <- roc(aki_vc_ml$AKI, as.numeric(error_rate_vc_rf))
print(roc_vc_rf)
ci(roc_vc_rf)
plot_vc_rf <- plot.roc(roc_vc_rf, col="blue",print.auc=FALSE, max.auc.polygon=FALSE,
                       print.thres=FALSE,auc.polygon=FALSE,legacy.axes=TRUE,add
= TRUE)

#------Support Vector Machine Regression -----#

install.packages("e1071")
library(e1071)

#creating svm model
```

```
aki_svm_tune <- tune(svm, factor(AKI)~., data=aki_dc_ml, ranges = list(cost
=c(1,10),gamma=c(0.1,0.5)))
summary(aki_svm_tune)
aki_svm_tune$best.parameters

aki_svm <- svm(factor(AKI)~.,data=aki_dc_ml, scale=F, gamma=0.5, cost=1)
summary(aki_svm)
aki_svm$fitted

#error rate
error_rate_dc_svm <- predict(aki_svm, data=aki_dc_ml[,-1])
mean(aki_dc_ml[,1]!= error_rate_dc_svm)

error_rate_vc_svm <- predict(aki_svm, newdata=aki_vc_ml[,-1])
mean(aki_vc_ml[,1]!= error_rate_vc_svm)

#ROC and AUC
roc_dc_svm <- roc(aki_dc_ml$AKI, as.numeric(error_rate_dc_svm))
print(roc_dc_svm)
ci(roc_dc_svm)
plot_dc_svm <- plot.roc(roc_dc_svm, col="red",print.auc=FALSE, max.auc.polygon=FALSE,
                        print.thres=FALSE,auc.polygon=FALSE,legacy.axes=TRUE)

roc_vc_svm <- roc(aki_vc_ml$AKI, as.numeric(error_rate_vc_svm))
print(roc_vc_svm)
ci(roc_vc_svm)
plot_vc_svm <- plot.roc(roc_vc_svm, col="blue",print.auc=FALSE, max.auc.polygon=FALSE,
                        print.thres=FALSE,auc.polygon=FALSE,legacy.axes=TRUE,add
= TRUE)

#------Naive Bayes Model -----#

install.packages("klaR")
library(klaR)

#creating naive bayes model
aki_nb_structure <- naive.bayes(aki_dc_ml, training = "AKI")
aki_nb <- bn.fit(aki_nb_structure, aki_dc_ml)

#error rate
error_rate_dc_nb <- predict(aki_nb, aki_dc_ml,prob = TRUE)
mean(aki_dc_ml[,1]!= error_rate_dc_nb)
table(aki_dc_ml$AKI,error_rate_dc_nb)
```

```
error_rate_vc_nb <- predict(aki_nb, aki_vc_ml,prob = TRUE)
mean(aki_vc_ml[,1]!= error_rate_vc_nb)
table(aki_vc_ml$AKI,error_rate_vc_nb)

#ROC and AUC
prob_dc_nb <- t(attr(error_rate_dc_nb, "prob"))[,2]
prob_vc_nb <- t(attr(error_rate_vc_nb, "prob"))[,2]

roc_dc_nb <- roc(aki_dc_ml$AKI, prob_dc_nb)
print(roc_dc_nb)
ci(roc_dc_nb)
plot_roc_dc_nb <- plot.roc(roc_dc_nb, col="red",print.auc=FALSE, max.auc.polygon=FALSE,
                           print.thres=FALSE,auc.polygon=FALSE,legacy.axes=TRUE)

roc_vc_nb <- roc(aki_vc_ml$AKI, prob_vc_nb)
print(roc_vc_nb)
ci(roc_vc_nb)
plot_roc_vc_nb <- plot.roc(roc_vc_nb, col="blue",print.auc=FALSE, max.auc.polygon=FALSE,
                           print.thres=FALSE,auc.polygon=FALSE,legacy.axes=TRUE,add
= TRUE)

#------Logistic Regression Model -----#

logit_form = as.formula(paste("aki_dc_ml$AKI~ ",paste(names(aki_dc_ml[,-
1]),collapse='+')))
aki_logit <- glm(logit_form,data=aki_dc_ml,family=binomial(link = "logit"))

#error rate
error_rate_dc_logit <- predict(aki_logit, data=aki_dc_ml[,-1],type="response")
error_rate_dc_logit2=ifelse(error_rate_dc_logit>0.5,1,0)
mean(aki_dc_ml[,1]!= error_rate_dc_logit2)
table(aki_dc_ml$AKI,error_rate_dc_logit2)

error_rate_vc_logit <- predict(aki_logit, newdata=aki_vc_ml[,-1],type="response")
error_rate_vc_logit2=ifelse(error_rate_vc_logit>0.5,1,0)
mean(aki_vc_ml[,1]!= error_rate_vc_logit2)
table(aki_vc_ml$AKI,error_rate_vc_logit2)

#ROC and AUC
roc_dc_logit <- roc(aki_dc_ml$AKI, error_rate_dc_logit)
print(roc_dc_logit)
ci(roc_dc_logit)
plot_dc_logit <- plot.roc(roc_dc_logit, col="red",print.auc=FALSE, max.auc.polygon=FALSE,
                          print.thres=FALSE,auc.polygon=FALSE,legacy.axes=TRUE)
```

```
roc_vc_logit <- roc(aki_vc_ml$AKI, error_rate_vc_logit)
print(roc_vc_logit)
ci(roc_vc_logit)
plot_vc_logit <- plot.roc(roc_vc_logit, col="blue",print.auc=FALSE, max.auc.polygon=FALSE,
                                   print.thres=FALSE,auc.polygon=FALSE,legacy.axes=TRUE,add
= TRUE)
```
